# Understanding Burn Injury Management: A Cross-Sectional Survey of Physicians Working in Northern Ontario Emergency Departments

**DOI:** 10.7759/cureus.114654

**Published:** 2026-08-17

**Authors:** Emily R Pynn, Sarah Hunt, Margarita Elloso, Tanya DeLyzer, Rabail Siddiqui, Sanjay Azad, David W Savage

**Affiliations:** 1 Department of Medicine, Northern Ontario School of Medicine (NOSM) University, Thunder Bay, CAN; 2 Department of Plastic and Reconstructive Surgery, Western University, London, CAN; 3 Department of Plastic and Reconstructive Surgery, Hamilton Health Sciences, Hamilton, CAN; 4 Department of Clinical Research Services, Thunder Bay Regional Health Sciences Centre, Thunder Bay, CAN; 5 Department of Plastic and Reconstructive Surgery, Northern Ontario School of Medicine (NOSM) University, Thunder Bay, CAN; 6 Department of Emergency Medicine, Northern Ontario School of Medicine (NOSM) University, Thunder Bay, CAN

**Keywords:** burn injury, burn management, emergency departments, plastic surgery, rural medicine

## Abstract

Purpose

In Northern Ontario, emergency physicians provide initial burn management, often without specialist support. This study evaluated the experience, practices, and resource limitations among Northern Ontario emergency physicians to identify gaps affecting burn care and specialist consultation.

Methods

An anonymous, cross-sectional REDCap survey was distributed by email to physicians providing emergency care across academic, community, and rural hospitals in Northern Ontario from May to August 2025. Physicians were recruited using convenience sampling through Northern Ontario School of Medicine (NOSM) University and hospital network contact lists. The survey assessed burn management knowledge and experience, clinical and referral practices, and perceived training and resource gaps. Responses were summarized using descriptive statistics, with categorical variables reported as frequencies and percentages.

Results

Fifty physicians responded; nearly half (48%, 24/50) practiced in rural settings, and most were family medicine-trained. Although 78% (39/50) reported moderate confidence in burn management, variability in knowledge and practice was evident. Twenty percent (10/50) incorrectly included superficial burns in total body surface area (TBSA) calculations, and only 66% (33/50) felt confident assessing burn depth. Initial management was consistent for analgesia, dressings, and tetanus prophylaxis but varied for debridement and fluid resuscitation; debridement of partial-thickness burns occurred only 42% (21/50) of the time. Most physicians (80%, 40/50) reported never having on-site plastic surgery coverage, and specialist consultations were sought primarily for transfer decisions and follow-up. Only 16% (8/50) reported consistent access to wound care. Most respondents were either unaware whether a burn directive existed at their site (52%, 26/50) or reported that no directive was in place (32%, 16/50). Nearly all participants (84%, 42/50) expressed interest in additional burn education.

Conclusions

Burn management in Northern Ontario emergency departments is highly variable, with knowledge gaps and limited specialist access potentially affecting outcomes. Regional standardization through a shared burn directive, combined with targeted physician education and expanded specialist access via teleburn or virtual consultation, may improve consistency of care across the region and in other settings facing similar constraints.

## Introduction

Thousands of Canadians sustain burn injuries every year [1]. The majority of these injuries are minor and can be effectively managed in ambulatory settings, such as the emergency department [2]. In Ontario, approximately 100 per 100,000 adults aged 18 to 64 years present annually to the emergency department with scald or contact burns, and 46 per 100,000 present with flame-related burns [3]. Burn presentations in the emergency department span a wide clinical spectrum, from minor injuries requiring basic first aid to severe cases requiring advanced medical and surgical interventions. Emergency providers play a central role in determining whether patients with burn injuries require transfer to a burn centre, hospital admission, or can be safely managed on an outpatient basis. When burns are not assessed or managed appropriately, patients can suffer complications, including infections, fluid and electrolyte imbalances, and long-term functional impairments [4]. Given the potential for serious sequelae and the complexity of burn care, coordinated early management is essential. However, the delivery of high-quality burn care is strongly influenced by broader health system factors, particularly in rural and remote settings.

Residents of Northern Ontario experience reduced access to health care services and poorer health outcomes compared with those living in more urban regions of the province [5]. Geographic remoteness and socioeconomic inequities contribute to these disparities, with Northern Ontario demonstrating higher baseline rates of poverty, food insecurity, smoking, and chronic disease, including diabetes and lung disease, compared with Southern Ontario [6-8]. Many residents live in rural or remote communities and must travel long distances to access tertiary and specialized care, a factor that has been associated with increased mortality and poorer outcomes, particularly in trauma settings [9,10]. Prehospital deaths are nearly twice as common in most rural areas, and delays of more than six hours to definitive trauma care may occur even after initial presentation at rural hospitals [10]. These challenges are further compounded by the vast geography and severe weather conditions common to northern regions [11]. Although much of this evidence is derived from trauma populations, similar structural barriers likely influence timely burn assessment and referral.

These systemic barriers have important implications for the acute management of injuries, including burns, in Northern Ontario. Limited access to specialized services, such as plastic surgeons, who provide most specialist burn care in Ontario, and wound care specialists, is a significant barrier to high-quality burn care. Variability in emergency physician training, inconsistent use of burn management guidelines, and logistical challenges related to patient transport and follow-up care may all also influence management decisions [11]. As a result, the standardization and consistency of burn care may differ across emergency care settings, potentially affecting early decision-making and referral consistency.

Given that emergency physicians provide most of the initial burn assessment, management, and disposition planning in Northern Ontario, understanding their experience, practice patterns, and access to resources is particularly important. Despite this, little is known about how emergency physicians in the region approach burn care in practice. This study aimed to characterize emergency physicians' experience, level of knowledge, and clinical practices related to burn management in emergency departments across Northern Ontario. Our goal was to describe physician demographics, evaluate knowledge and expertise, and summarize the availability of referral and specialist services. By understanding these factors, we sought to characterize the unique challenges encountered by health care providers in the region and to support the development of measures and educational tools that improve burn management consistency, support equitable access to specialist consultation, and strengthen regional educational initiatives.

## Materials and methods

Data were collected from Northern Ontario physicians working in emergency departments via a cross-sectional survey. The survey consisted of multiple-choice, Likert-scale, and open-ended questions. The survey gathered data on participants' professional and demographic characteristics, their knowledge and experience in managing burn injuries, their referral and follow-up practices, and their perceived training needs and resource constraints (Appendix 1). The survey was reviewed by four physicians (plastic surgeons and emergency physicians) for content validity and pilot tested in REDCap by two physicians to assess clarity and functionality prior to distribution.

The target population consisted of physicians providing emergency care who work in various hospital types (academic centres, community hospitals, and rural hospitals) in Northern Ontario. The sampling frame was constructed using contact lists from Northern Ontario School of Medicine (NOSM) University and hospital networks. A convenience sampling method was used to reach physicians via email because of the accessibility of email lists and the goal of reaching a broad spectrum of practitioners.

Physicians practicing emergency medicine in Northern Ontario received the survey electronically through email. The survey was administered via REDCap, a secure platform for data collection. The survey was completed online by participants between May 1 and August 1, 2025. Two standardized reminders were sent to boost response rates.

Informed consent was obtained from all participants, ensuring confidentiality and data protection. Participants were assured of anonymity, and all responses were securely stored on password-protected, institutionally approved devices. The study was approved by the Institutional Research Ethics Board at Lakehead University (No. 1470909), and all data were collected in compliance with institutional ethical guidelines.

Descriptive statistics were used to summarize the survey responses. Categorical variables were summarized as frequencies and percentages.

## Results

Demographics

A total of 50 physicians working in Northern Ontario emergency departments fully completed the survey. Approximately 120 physicians were estimated to be eligible, yielding an estimated response rate of 42% (50/120). However, because no centralized registry exists across participating sites, the denominator and resulting response proportion should be interpreted as approximate. Certification among respondents included Certification of the College of Family Physicians of Canada in Emergency Medicine (CCFP(EM)) (54%, 27/50), Certification of the College of Family Physicians of Canada (CCFP) (34%, 17/50), and Fellowship of the Royal College of Physicians of Canada (FRCPC) (12%, 6/50). In Canada, CCFP(EM) physicians complete a two-year family medicine residency and an additional one-year emergency medicine training program, whereas FRCPC physicians complete a five-year Royal College-accredited emergency medicine residency. CCFP physicians complete a two-year family medicine residency and may practice emergency medicine, particularly in rural settings. Over half of the physicians (54%, 27/50) reported fewer than 10 years of clinical experience specifically in Northern Ontario, and nearly half (48%, 24/50) worked in rural hospitals. The detailed demographics of the survey population are presented in Table 1.

**Table 1:** Demographics ^a^CCFP = Certification in the College of Family Physicians of Canada; ^b^CCFP (EM) = Certification in the College of Family Physicians of Canada with a Certificate of Added Competence in Emergency Medicine;​​​​​​​ ^c^FRCPC = Fellow of the Royal College of Physicians of Canada

Variables	N (%)
Gender	
Male	24 (48)
Female	24 (48)
Unspecified	2 (4)
Age	
20-29	1 (2)
30-39	18 (36)
40-49	18 (36)
50-59	9 (18)
60 or above	4 (8)
Certification	
CCFP^a^	17 (34)
CCFP (EM)^b^	27 (54)
FRCPC^c^	6 (12)
Experience in Emergency Medicine (Years)	
0-9	24 (48)
10-19	19 (38)
20-29	4 (8)
30-39	3 (6)
Experience in Northern Ontario (Years)	
0-9	27 (54)
10-19	17 (34)
20-29	4 (8)
30-39	2 (4)
Type of Hospital	
Community Hospital	9 (18)
Rural Hospital	24 (48)
Academic Health Sciences Centre	17 (34)

Education and experience

Most respondents had received education on burn management through postgraduate medical training (76%, 38/50). While 48% (24/50) of physicians reported seeing only one to four burn patients per year, more than one-third (34%, 17/50) encountered five to nine cases annually. Thermal burns were the most common type of burn injury encountered (98%, 49/50). Most participants felt somewhat or fairly confident (78%, 39/50) in managing burn injuries and felt slightly or somewhat confident (66%, 33/50) in assessing burn depth. Additional details on education sources and physician experience are presented in Table 2.

**Table 2:** Education and experience *Respondents could select more than one response; percentages do not sum to 100%.

Variables	N (%)
Education Source*	
Undergraduate Medical Education (UME)	36 (72)
Postgraduate Medical Education (PGME)	38 (76)
Continuing Medical Education (CME)	29 (58)
Burn Patient Volume (per year)	
Rarely (1-4 patients/year)	24 (48)
Often (5-9 patients/year)	17 (34)
Frequently (≥10 patients/year)	9 (18)
Most Common Burn Injury Encountered	
Thermal	49 (98)
Chemical	1 (2)
Confidence Managing Burn Injuries	
Not confident at all	2 (4)
Slightly confident	6 (12)
Somewhat confident	21 (42)
Fairly confident	18 (36)
Extremely confident	3 (6)
Confidence Assessing Burn Depth	
Not confident at all	2 (4)
Slightly confident	11 (22)
Somewhat confident	22 (44)
Fairly confident	14 (28)
Extremely confident	1 (2)

Practice patterns

Regarding burn management, 20% (10/50) of respondents included superficial, partial-thickness, and full-thickness burns in their total body surface area (TBSA) calculations, whereas 72% (36/50) accurately limited their calculations to partial- and full-thickness burns. Initial management varied among the respondents, and the most frequent initial interventions were pain control (100%, 50/50), wound dressing (96%, 48/50), and tetanus prophylaxis (92%, 46/50). Non-adherent dressings were the most frequent choice for wound dressing (96%, 48/50), with fewer respondents using silver-based products (20%, 10/50) and only 56% (28/50) applying antimicrobial ointment. Initiation of fluids began at 10% TBSA or greater in 38% (19/50) of respondents. Most providers used lactated Ringer solution (70%, 35/50) and followed the modified Brooke formula (54%, 27/50) or Parkland formula (38%, 19/50) to determine the volume of intravenous fluid. Only 42% (21/50) of respondents reported debriding partial-thickness blisters often or sometimes. Regarding comfort with debridement, fewer than half (42%, 21/50) reported feeling somewhat comfortable, and 24% (12/50) reported feeling somewhat uncomfortable. Over half of the respondents (64%, 32/50) consulted plastic surgeons at least sometimes, primarily for transfer advice (80%, 40/50) or arranging follow-up (70%, 35/50). Referral practices to specialized burn centres varied; common referral criteria included burns in critical locations (96%, 48/50), full-thickness injuries (82%, 41/50), or those involving 10% or more TBSA (72%, 36/50). Detailed practice patterns for burn assessment, wound management, and referral are presented in Table 3.

**Table 3:** Practice patterns *Respondents could select more than one response; percentages do not sum to 100%. TBSA: total body surface area

Variables	N (%)
TBSA Calculation Depths Included*	
Superficial	10 (20)
Partial-thickness	48 (96)
Full-thickness	45 (90)
Initial Management Steps*	
Pain management	50 (100)
Fluid resuscitation	46 (92)
Irrigation	34 (68)
Debridement	10 (20)
Wound dressing	48 (96)
Tetanus prophylaxis	46 (92)
Initial Wound Care Strategies*	
Leave open to air	6 (12)
Apply a dry dressing	3 (6)
Apply a non-adherent dressing	48 (96)
Apply a silver-based cream	7 (14)
Apply a silver-based dressing	10 (20)
Apply antimicrobial ointment	28 (56)
TBSA Threshold to Initiate Fluids	
≥10% TBSA	19 (38)
≥15% TBSA	13 (26)
≥20% TBSA	14 (28)
≥25% TBSA	2 (4)
All burn patients	2 (4)
Preferred Fluid for Resuscitation	
Lactated Ringer solution	35 (70)
Normal saline	15 (30)
Fluid Initiation Method	
As per Parkland formula	19 (38)
As per Modified Brooke formula	27 (54)
Crystalloid bolus wide open	4 (8)
Debridement Frequency of Partial Thickness Burns	
Never	9 (18)
Rarely	20 (40)
Sometimes	14 (28)
Often	7 (14)
Debridement Comfort Level	
Extremely uncomfortable	1 (2)
Very uncomfortable	5 (10)
Somewhat uncomfortable	12 (24)
Somewhat comfortable	21 (42)
Very comfortable	10 (20)
Extremely comfortable	1 (2)
Frequency of Plastic Surgery Consultation	
Never	1 (2)
Rarely	14 (28)
Sometimes	18 (36)
Often	14 (28)
Not applicable	3 (6)
Reasons for Plastic Surgery Consultation*	
Consideration of transfer to burn centre	40 (80)
Arrange reliable follow-up	35 (70)
Wound management advice	30 (60)
Formal assessment and opinion	27 (54)
Consideration of surgery	23 (46)
Consideration of admission	18 (36)
Initiate wound care	12 (24)
Reasons for Referral to Burn Centres*	
Location of burn (face, genitals, hands, feet, perineum, or major joints)	48 (96)
Full-thickness burn	41 (82)
Age of the patient	39 (78)
TBSA >10%	36 (72)
Inhalation injury	35 (70)
Comorbidities	26 (52)

Resource limitations

**Figure 1:**
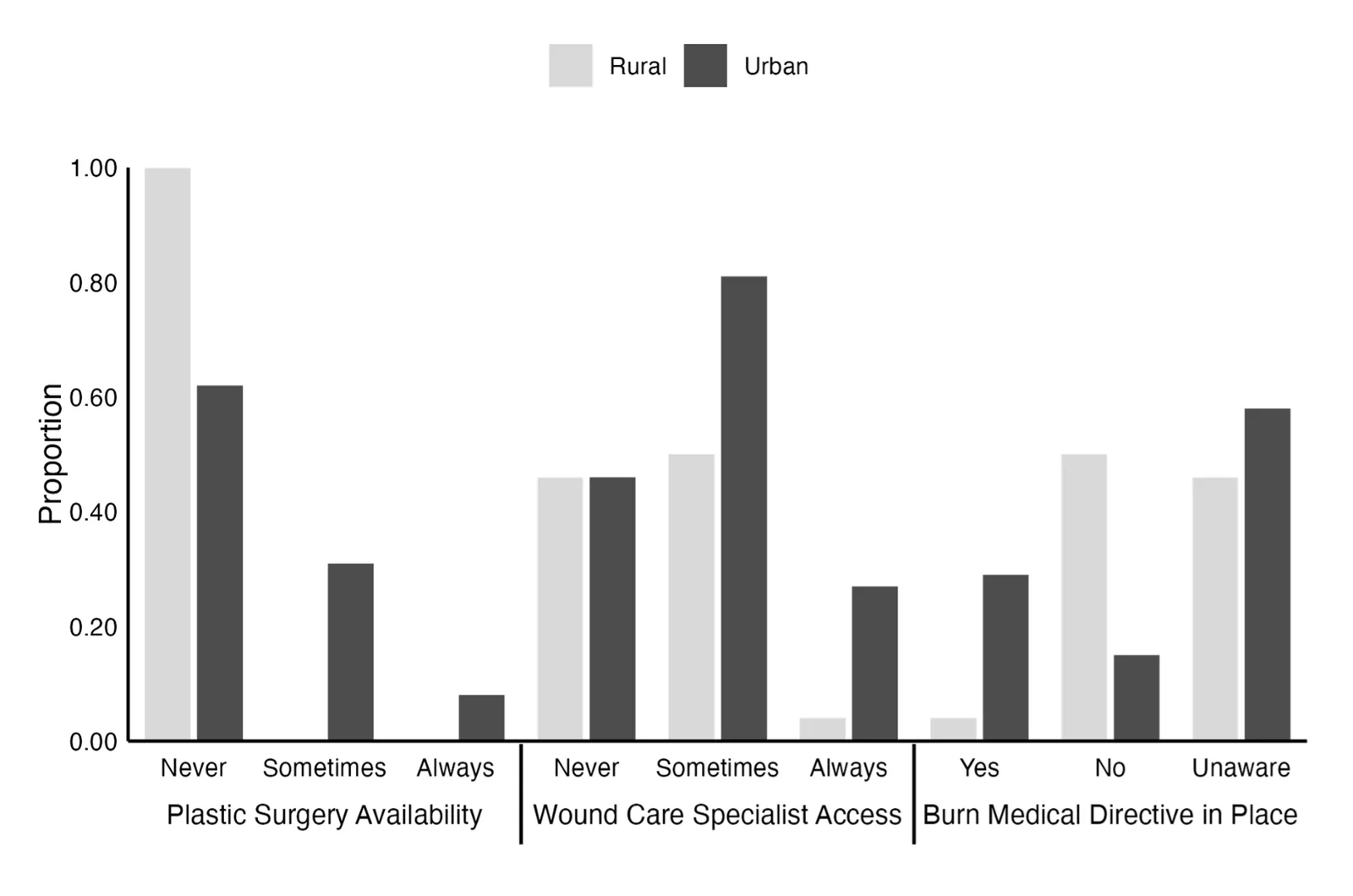
Availability of burn-related resources in rural and urban emergency departments

Access to specialist consultation was limited, and most physicians (80%, 40/50) reported never having plastic surgery coverage on site. Access to wound care specialists was inconsistent, with only 16% (8/50) reporting that such services were always available. Limited specialist access was identified as the most significant barrier to optimal burn care (90%, 45/50), followed by complexity of cases (64%, 32/50) and limited knowledge (40%, 20/50). Equipment adequacy was a concern, with only 38% (19/50) reporting sufficient resources. Most respondents were either unaware of whether a burn directive existed at their site (52%, 26/50) or reported that no directive was in place (32%, 16/50), although half of physicians (50%, 25/50) considered such a directive important. Rural sites consistently reported lower access to specialist support and standardized resources than urban centres, as detailed in Figure 1.

Educational needs

Physicians were asked to report their perceived educational needs and preferred formats for additional burn management training. Almost all physicians (84%, 42/50) expressed interest in further training, with online learning (62%, 31/50) and in-person workshops (44%, 22/50) most frequently identified as the preferred formats.

## Discussion

This study provides insight into burn management practices, resource availability, and perceived educational needs among emergency physicians practicing in Northern Ontario. Rather than a set of isolated deficits, the pattern across our findings - variable assessment and early management, limited access to expert support, and an interest in continuing education - points to a burn care system that is not currently structured to support consistent decision-making at the point of care. These challenges appear to reflect the realities of delivering burn care across a geographically vast region with limited specialist resources.

Although most respondents reported moderate confidence managing burn injuries, several foundational aspects of burn care revealed important gaps. Physicians were generally comfortable with routine emergency interventions, such as pain management, tetanus prophylaxis, and basic wound dressings, which were also the most consistently reported elements of initial burn management [12,13]. In contrast, lower confidence and greater variation were reported for more specific burn management interventions, such as burn depth assessment, TBSA estimation, fluid resuscitation decisions, and procedures such as blister debridement. These are precisely the steps that determine whether a patient is triaged, resuscitated, and referred appropriately; thus, gaps concentrated here carry outsized weight for downstream decision-making. These skills require more specialized knowledge and clinical judgment that may be difficult to maintain in low-volume settings.

A subset of respondents reported including superficial burns in TBSA calculations, which contrasts with standard practice that defines TBSA using validated tools such as the Rule of Nines, Lund-Browder chart, or Palmer method and excludes superficial burns [14]. This discrepancy matters beyond documentation accuracy, as referring hospitals and providers tend to overestimate burn area, at rates up to 30 times higher than the rate of underestimation [15]. The area of minor burns (<20% TBSA) is frequently overestimated, resulting in excessive fluid resuscitation, whereas the area of more extensive burns (>20% TBSA) is typically underestimated and thus underresuscitated [15]. Overresuscitation is associated with complications such as fluid overload, abdominal compartment syndrome, pulmonary edema, and hyperchloremic metabolic acidosis [15,16]. Conversely, inadequate fluid resuscitation could result in circulatory collapse and renal failure, ultimately progressing to burn shock, a combination of distributive, cardiogenic, and hypovolemic shock [16]. Although use of established resuscitation formulas was common, ongoing reliance on the Parkland formula alongside the modified Brooke formula, the latter of which is associated with lower resuscitation volumes, suggests continued variability in burn resuscitation practices [17]. This variability has been associated with adverse clinical outcomes [17]. In a system without on-site burn specialists to catch the discrepancies before transfer, a single standardized resuscitation protocol may provide an important safeguard against variation in care.

Variation was also evident in early wound care practices. Although most respondents reported appropriate use of non-adherent dressings, slightly more than half applied topical antimicrobial agents, despite clinical burn care recommendations to prevent wound infection and support early wound care [18-20]. In addition, fewer than half reported routinely debriding partial-thickness blisters, despite the important role debridement plays in facilitating accurate wound assessment and may reduce infection risk and delayed healing [20,21]. Early burn debridement is vital to the overall survival and outcome of burn patients [21]. Comfort with debridement appeared to differ across certification types in our sample, though given the descriptive design of this study, this observation was not formally tested and should be read as hypothesis-generating rather than confirmatory. More broadly, the findings may suggest that differences in training and clinical exposure may influence comfort with burn-specific procedures, potentially more so than years in practice alone, particularly among physicians working in rural emergency departments.

Nearly half of physicians managed only one to four burn cases per year, and fewer than half encountered burns frequently enough to maintain regular exposure to higher-acuity injuries. This highlights the challenge of maintaining competency for a low-frequency, potentially high-acuity clinical condition. This challenge has been described in rural trauma care, where lower patient volumes and limited procedural exposure create difficulty for skill retention and knowledge maintenance [22]. Variability in burn management practices was observed across all experience levels in our sample, suggesting that years in practice alone do not necessarily translate into proficiency in burn care without ongoing reinforcement. This finding aligns with findings from rural trauma care, where no significant association has been reported between overall comfort with trauma procedures and years in practice or community size [23].

Given the noted variability, access to specialist support becomes particularly consequential; without a backup, individual knowledge gaps translate more directly into patient-level consequences. Most respondents reported no on-site plastic surgery coverage and inconsistent access to wound care services, with reported disparities between rural and urban centres. These findings align with prior literature describing reduced access to specialized services, educational resources, and burn care supplies in rural and northern regions of Ontario, which can contribute to inequities in care [23]. Similar challenges have been documented in rural trauma systems, where delays in access to definitive care are associated with poorer outcomes [10]. The implication is that specialist access in Northern Ontario is a structural constraint that may limit how much any single intervention, whether education or protocols, can accomplish on its own.

Despite these constraints, referral practices were largely consistent with American Burn Association guidelines [24]. Most respondents appropriately identified burns involving critical areas, full-thickness injuries, or larger TBSA as indications for referral. This finding suggests that emergency physicians are generally able to recognize injuries requiring a higher level of care, even in the absence of local burn directives. However, the lack of standardized burn-specific protocols may contribute to uncertainty in borderline cases, particularly in low-volume settings.

Future directions

Nearly all respondents expressed interest in additional education related to burn management, with online learning and in-person workshops identified as preferred formats. These findings highlight an opportunity for targeted burn education tailored to emergency physicians practicing in Northern Ontario. Online continuing professional development programs may be particularly well suited to geographically dispersed regions and have been shown to improve knowledge and performance among rural health care providers [25,26].

Telemedicine also represents a promising strategy for supporting burn care in resource-limited settings. Prior studies have demonstrated that real-time videoconferencing during burn assessments can improve clinical decision-making and outcomes in rural areas [27]. In Northern Ontario, established intensive care unit-based video outreach programs in Thunder Bay and Sudbury support rural patient care by enabling intensivists, critical care nurses, and respiratory therapists to provide real-time guidance on resuscitation, triage, ongoing management, and transfer decision-making [28]. Expanding similar tele-video consultation models to include burn centres may provide timely expert input and support early management decisions.

A dedicated Teleburn consultation program is available through Southern Ontario; however, the extent to which emergency physicians in Northern Ontario use this service remains unclear [29]. Evaluating physicians' awareness, patterns of use, and perceived barriers to access of this service represents a feasible and important next step to inform strategies aimed at improving integration of Teleburn services within Northern Ontario emergency departments.

The development of a regional burn medical directive would also be beneficial for promoting standardized assessment, resuscitation, and referral practices across Northern Ontario emergency departments. Future research could examine how such directives are adopted in practice and how they influence early management and referral decisions. Similar studies in other regions with large rural catchment areas may help determine whether comparable gaps exist and inform broader provincial approaches to burn care education and system planning.

Study limitations

Several limitations of this study must be acknowledged. First, the sample size was modest, and the lack of a centralized registry of eligible emergency physicians prevented calculation of a precise response rate. Furthermore, the use of a convenience sample may limit the generalizability of the findings to all providers in the region and may introduce selection bias, because those with a particular interest, experience, or lack thereof in burn care may have been more likely to respond. The study population may overrepresent providers with existing awareness of burn care deficiencies. In addition, reliance on self-reported data is subject to recall and social desirability bias, which may have resulted in an overestimation of respondents' comfort with aspects of burn assessment and management.

Despite these limitations, the inclusion of respondents from a range of urban, community, and rural emergency departments provides valuable insight into real-world burn management practices in a geographically remote region that is underrepresented in the burn care literature.

## Conclusions

Burn injuries present an ongoing clinical challenge in Northern Ontario emergency departments, where access to specialized burn care is limited by geography and resource constraints. The variability observed in burn assessment, resuscitation, and early wound care in this study is therefore better understood as a system-level gap rather than a series of isolated deficits, reflecting limited access to standardized tools and specialist support at the point of care.

Addressing this gap will require more than education alone. Regional standardization through a shared burn medical directive, paired with targeted physician education and expanded specialist access through teleburn and virtual consultation, may offer a practical approach to improving referral quality and consistency of care across Northern Ontario. The challenge of managing a low-frequency, high-acuity condition across a vast, resource-limited catchment is not unique to Northern Ontario, and this combined approach may serve as a practical model for other rural and remote health systems facing similar constraints.

## Appendices

Appendix 1

**Table 4:** ED burn management survey instrument

#	Question	Response Options
1	Which option best describes your gender? (Select all that apply)	Gender fluid; I identify as: ___; Man; Non-binary; Prefer not to answer; Queer; Two-spirit; Woman
2	What is your age (years)?	20-29; 30-39; 40-49; 50-59; 60 or above; Prefer not to reply
3	What is your certification?	CCFP; CCFP (EM); FRCPC; Other
4	How many years of experience do you have in emergency medicine?	0-9 years; 10-19 years; 20-29 years; 30-39 years; 40 or more years
5	How many years have you been practicing in Northern Ontario?	0-9 years; 10-19 years; 20-29 years; 30-39 years; 40 or more years
6	What type of hospital do you practice?	Academic Health Sciences Center; Community Hospital; Rural Hospital; Other
7	Where did you receive your education on burn management? (Select all that apply)	Undergraduate medical education; Post graduate medical education; Continuing education and professional development; Online resources; Other
8	What is the most common type of burn injury you encounter?	Thermal (flame, contact, scald); Chemical; Electrical; Radiation; Other
9	How often do you encounter burn injuries?	Never; Rarely (1-4 patients/year); Often (5-9 patients/year); Frequently (≥10 patients/year)
10	How confident are you in managing burn injuries?	Extremely confident; Fairly confident; Somewhat confident; Slightly confident; Not confident at all
11	What is your primary method to assess total body surface area affected by burns?	Rule of Nines; Lund and Browder chart; Palmar method; Other
12	How familiar are you with the following burn assessment tools? a) Rule of Nines b) Palmar Method c) Lund and Browder chart	Very familiar; Familiar; Somewhat familiar; Unfamiliar; Very unfamiliar (rated for each tool)
13	What criteria do you use to assess the severity of a burn injury? (Select all that apply)	Total body surface area; Skin appearance; Inhalation injury; Age of the patient; Location of burn; Cause of burn; Other
14	How confident are you in assessing burn depth?	Extremely confident; Fairly confident; Somewhat confident; Slightly confident; Not confident at all
15	What burn depths do you include in your TBSA calculation? (Select all that apply)	Superficial; Partial thickness; Full thickness
16	What initial management steps do you typically follow for burn injuries? (Select all that apply)	Administer tetanus prophylaxis; Wound dressing; Irrigate wound; Pain management; Fluid resuscitation; Debridement; Other
17	When do you initiate fluid resuscitation?	Never; All burns; 10% TBSA or greater; 15% TBSA or greater; 20% TBSA or greater; 25% TBSA or greater; 30% TBSA or greater; Other
18	How do you initiate fluid management?	Crystalloid boluses wide open; Crystalloid boluses at a rate of 1L/hour; As per Parkland Formula (4mL/kg/TBSA); As per Modified Brooke/Modified Parkland Formula (2mL/kg/TBSA); At a maintenance rate; N/A
19	What is your preferred fluid for resuscitation?	Ringer lactate; Normal saline; D5 normal saline; N/A
20	How often do you debride blisters associated with partial thickness burns?	Always; Often; Sometimes; Rarely; Never
21	What is your level of comfort debriding blisters associated with partial thickness burns?	Extremely comfortable; Very comfortable; Somewhat comfortable; Somewhat uncomfortable; Very uncomfortable; Extremely uncomfortable
22	What are your most commonly used wound care strategies for burns? (Select all that apply)	Leave open to air; Apply silver-based cream; Apply silver-based dressings; Apply antimicrobial ointment; Apply non-adherent dressings; Apply dry dressings; Other
23	How comfortable are you in initiating and advising patients on pain control strategies for burns?	Extremely comfortable; Very comfortable; Somewhat comfortable; Somewhat uncomfortable; Very uncomfortable; Extremely uncomfortable
24	Is there a plastic surgeon available for bedside consultations at your facility?	Always; Sometimes; Never
25	What frequency do you consult with the plastic surgeon for burn injuries?	Always; Often; Sometimes; Rarely; Never; N/A
26	What are the primary reasons for requesting a consultation with a plastic surgeon in your practice? (Select all that apply)	Formal assessment and opinion; Wound management advice; Initiating wound care; Consideration of admission; Consideration of surgery; Consideration of patient transfer to burn centre; Arranging reliable follow-up; Other
27	Do you have access to wound care specialists for burn care follow-up?	Always; Sometimes; Never
28	How often do you refer burn injury patients to specialized burn centres?	Always; Often; Sometimes; Rarely; Never; N/A
29	When do you refer to specialized burn centres? (Select all that apply)	>10% Total Body Surface Area; Age of the patient; Comorbidities; Full thickness burn; Inhalation injury; Location of burn (Face, genitals, hands, feet, perineum, or major joints); Other
30	What factors impact on your ability to manage burn injuries in Northern Ontario? (Select all that apply)	Specialist availability; Availability of necessary equipment; Time constraints in emergency room; Complexity of burns; Limitation in knowledge; Psychosocial supports for patients; Other
31	How do you perceive the availability of resources (e.g., equipment, staffing) for managing burn injuries in your hospital?	Extremely adequate; Very adequate; Adequate; Somewhat adequate; Not adequate
32	Are there medical directives currently in place for managing burn injuries at your hospital?	Yes; No; Unaware
33	How important do you think a burn injury medical directive is for your emergency department?	Very important; Fairly important; Important; Somewhat important; Not important; N/A
34	In your opinion, having a base knowledge about burn injury management is:	Very important; Fairly important; Important; Somewhat important; Not important
35	Would you like to receive more training to improve your ability to manage burn injuries?	Yes; No
36	What format of additional training would you prefer? (Select all that apply)	Online resources; In-person workshops; Webinars; Printed materials; Mobile apps; Other
37	Do you have any further comments on burn injury management in Northern Ontario? (Please specify)	Open-ended response
